# Unintended Consequences of Incentive Provision for Behaviour Change and Maintenance around Childbirth

**DOI:** 10.1371/journal.pone.0111322

**Published:** 2014-10-30

**Authors:** Gill Thomson, Heather Morgan, Nicola Crossland, Linda Bauld, Fiona Dykes, Pat Hoddinott, on behalf of the BIBS team

**Affiliations:** 1 Maternal and Infant Nutrition & Nurture Unit (MAINN), School of Health, University of Central Lancashire, Preston, Lancashire, England; 2 Health Services Research Unit, University of Aberdeen, Aberdeen, Scotland; 3 Nursing, Midwifery and Allied Health Professions Research Unit, University of Stirling, Stirling, Scotland; TNO, Netherlands

## Abstract

Financial (positive or negative) and non-financial incentives or rewards are increasingly used in attempts to influence health behaviours. While unintended consequences of incentive provision are discussed in the literature, evidence syntheses did not identify any primary research with the aim of investigating unintended consequences of incentive interventions for lifestyle behaviour change. Our objective was to investigate perceived positive and negative unintended consequences of incentive provision for a shortlist of seven promising incentive strategies for smoking cessation in pregnancy and breastfeeding. A multi-disciplinary, mixed-methods approach included involving two service-user mother and baby groups from disadvantaged areas with experience of the target behaviours as study co-investigators. Systematic reviews informed the shortlist of incentive strategies. Qualitative semi-structured interviews and a web-based survey of health professionals asked open questions on positive and negative consequences of incentives. The participants from three UK regions were a diverse sample with and without direct experience of incentive interventions: 88 pregnant women/recent mothers/partners/family members; 53 service providers; 24 experts/decision makers and interactive discussions with 63 conference attendees. Maternity and early years health professionals (n = 497) including doctors, midwives, health visitors, public health and related staff participated in the survey. Qualitative analysis identified ethical, political, cultural, social and psychological implications of incentive delivery at population and individual levels. Four key themes emerged: how incentives can address or create inequalities; enhance or diminish intrinsic motivation and wellbeing; have a positive or negative effect on relationships with others within personal networks or health providers; and can impact on health systems and resources by raising awareness and directing service delivery, but may be detrimental to other health care areas. Financial incentives are controversial and generated emotive and oppositional responses. The planning, design and delivery of future incentive interventions should evaluate unexpected consequences to inform the evidence for effectiveness, cost-effectiveness and future implementation.

## Introduction

There has been growing international academic and policy interest in the use of financial incentives to change health behaviours [1]–[3]. Similarly, there is evidence, particularly from the UK Quality and Outcomes Framework, that financial incentives to care providers can change health professional behaviour resulting in improved health outcomes; however to date disease rather than ‘lifestyle’ behaviour outcomes have been the focus [4], [5]. Literature on the mechanisms of action of incentive interventions suggests that incentives can “crowd out” intrinsic motivation [6]–[8] when financial incentives are viewed as paternalistic or undermining autonomy when recipients are ‘told what to do’ [9].

‘Unintended consequences’ is a key area of contention within incentive delivery. The phrase was first coined by Merton [10] and refers to outcomes other than the ones intended by a purposeful action. The purposeful actions intended by financial incentives in health care are changes in behaviour (at citizen, patient, healthcare provider or organisation level) which lead to evidence of improved health outcomes (effectiveness and/or cost-effectiveness). However, negative consequences have been reported, particularly by the media which describe incentives as potentially coercive or encouraging unhealthy behaviours or game playing to ensure eligibility [11]–[13], generating public and tax payers’ debate about the appropriateness of incentives, particularly in countries with state-funded health services.

Systematic reviews conducted as part of the BIBS (Benefits of Incentives for Breastfeeding and Smoking Cessation) study, which aimed to inform the design of incentive intervention trials for smoking cessation in pregnancy and breastfeeding, provide the background to this paper and are reported in full elsewhere [14], [15]. Incentives were defined as ‘financial (positive or negative) and non-financial tangible incentives or rewards, such as free or reduced cost items or services that have a monetary or an exchange value’ [14]. Systematic reviews investigated i) the evidence for the effectiveness of incentive interventions delivered within or outside the health service, to: a) individuals, families or b) organisations that aim to increase and sustain smoking cessation and breastfeeding [14] and included a narrative review of qualitative and process evaluation data; ii) a narrative synthesis of qualitative reviews reporting barriers and facilitators to smoking cessation and breastfeeding; iii) a scoping narrative review of reviews of the effectiveness of financial incentives for other lifestyle behaviours relevant to women of childbearing age.

Data relating to the consequences of incentive interventions (intended and unintended) were extracted and analysed in these reviews. In summary, the evidence syntheses did not identify any primary research with the aim of investigating unintended consequences of incentive interventions for lifestyle behaviour change. Positive and negative unintended consequences of incentive provision were sometimes referred to within the discussion section of the papers. Gaming and cheating were reported as a concern for incentives to influence smoking cessation, particularly in high risk populations, (e.g. the vulnerable/those with chaotic life styles) [16]–[18]. They may be considered ‘unfair’ for those who make healthy choices [19], [20]. While financial incentives to health care providers are considered to narrow health inequalities between the most and least deprived populations [4], [21], a review of the evidence for incentive schemes to encourage positive health and social behaviours in young people identified that incentives targeted some groups and not others and this was perceived as unethical and inequitable [22]. This review [22] and a smoking cessation in pregnancy incentive intervention delivered through pharmacies [23] suggest that incentive programmes may not achieve their intended demographic reach, with more advantaged groups benefiting from the programme. Furthermore, if behaviour change is not achieved, self-esteem may be reduced and incentives that are negatively perceived can cause harm, such as undesirable peer pressure and bullying [22], [24].

Incentives to providers have been reported to inadvertently promote unethical ‘gaming’ behaviour, through distortion, manipulation or concealment of data [25], [26]. While incentives can improve the performance and cost-effectiveness of care on targeted conditions [4], large scale/complex incentive interventions are administratively labour and resource-intensive [22], [27]. Concerns have also been raised about the neglect of non-incentivised conditions or patients for whom the conditional quality target for providers to receive the incentive is more difficult to achieve [4], [26]. Evidence suggests that care provider efforts tend to wane once the target has been achieved [4] and that improvements occur at the fastest rate in the first year of a programme and subsequently return to the pre-intervention rates of improvement [4], [21]. Incentives can improve teamwork, enhance specialist skills [4], [18], [28] and facilitate connections between providers and consumers [29]. However other studies report reductions in person-centeredness, patient satisfaction and continuity of care [4], [30] as well as expectations for financial reimbursement across other areas of health care [26].

In this paper, we report on mixed methods primary research on the positive and negative unintended consequences of financial incentive provision (to consumers and providers) where the intended consequences are defined as smoking cessation and breastfeeding behaviour change and maintenance around childbirth and the unintended consequences concern outcomes other than those intended. ‘Consumers’ refer to the target population whose behaviour the incentive aims to change. Some, but not all, will be health service patients. ‘Providers’ refer to any staff, services or organisations which have a role in supporting women to stop smoking in pregnancy and/or to breastfeed after birth.

## Materials and Methods

### Ethics

Full ethical approval for this study, including service user involvement, was obtained from the North of Scotland Research Ethics Committee (NOSRES, reference number: 12/NS/0041, 12th April 2012), and subsequent permissions were granted locally by Research and Development, NHS Grampian (24th April 2012) and the BUSH (Built & Natural Environment, Sport and Health) Ethics Committee, University of Central Lancashire (BUSH064, 8th May 2012). Amendments were submitted to NOSRES. AM01 to cover the amendments required by BUSH (approved 10th May 2012); AM02 to allow us to use a flyer for recruiting health professionals at conferences and an information leaflet designed for partners/family/friends (approved 6th December 2012) and AM03 to gain ethical approval for the contents of the general public and health professionals survey (approved 17th April 2013). Participants who took part in the interviews, focus groups or interactive discussions provided written (for face to face interviews/focus groups) or verbal (for telephone interviews, interactive discussions) informed consent to participate in this study. Survey respondents were not requested to complete a consent form, rather consent was implied by their participation. All consent procedures received ethics approval.

### Study design

Three evidence syntheses described above were integrated with primary qualitative and survey research to investigate diverse perspectives on incentives for smoking cessation in pregnancy and breastfeeding using a multi-disciplinary, mixed methods approach. Researchers regularly engaged two mother-and-baby groups in disadvantaged areas (study co-applicants) who provided diverse, hard-to-reach service-user involvement.

### Qualitative data

#### Setting and participants

Three settings (Lancashire, Glasgow and Aberdeen) were purposively selected for their diverse socio-demographic characteristics and their different incentive cultures for smoking cessation in pregnancy and breastfeeding (Table 1 provides an overview of the demographics and characteristics of the three selected sites). A third sector incentive scheme designed for young parents had been implemented in Aberdeen. Health service incentive schemes for both target behaviours had been operating in Lancashire. In Glasgow, a concurrent health service smoking cessation Phase II incentive trial was being undertaken with a qualitative process evaluation [34].

**Table 1:** Overview of recruitment sites.

**Aberdeenshire** has a mixed urban/town/rural population, with partners absent for long spells working offshore in fishing and the oil industry and pockets of affluence and deprivation. In Grampian in 2012, 14.5% of women were reported as current smokers at antenatal booking and 13.5% at 10–14 days after birth [31]. In 2011/2, 58.4% of babies were being given some breast milk at 10–14 days, with 45.4% still receiving some breast milk at 6–8 weeks after birth [31]. *Incentive culture*: It has the highest proportion in Scotland (71%) of smoking cessation services to pregnant women delivered through community pharmacists, who receive payments per person registering for smoking cessation support and for data collection. In discussions between PH and providers in primary care and maternity services, many managers and practitioners are resistant to providing financial incentives to patients following adverse media publicity about a smoking cessation incentive scheme in neighbouring Tayside. Our co-applicant mother and baby group is an example of a partnership community development project which has raised money from local businesses to provide non-financial incentives (a crèche and subsidised cafe).
**Glasgow** has an urban multi-cultural population with a wide socio-demographic range from affluence to large areas of extreme disadvantage. 50% of households are in areas of the highest material deprivation compared with 20% for Scotland as a whole. In 2012, 18.3% of women living in Greater Glasgow and Clyde were reported as current smokers at antenatal booking and 15.3% at 10–14 days after birth [32]. In 2011/2, 43.4% of babies were being given some breast milk at 10–14 days, with 33.9% still receiving some breast milk at 6–8 weeks after birth [33]. *Incentive culture:* The CPIT trial started in June 2011 and includes a qualitative element examining how incentives are perceived by recipients and providers [34].
**Lancashire** has a mixed urban, small town and rural population with a wide socio-demographic range. For 2007 Indices of Deprivation, six local districts (including Blackpool) are ranked within the top 50 in England and some towns have up to 35% of births to women of South Asian origin. Blackpool has one of the highest rates of teenage pregnancy, one of the lowest breastfeeding initiation rates in 2012 (56% compared to 74% for England) and babies still breastfed at 6–8 weeks (24% compared to 47% for England) [35]. Whilst smoking rates vary across the region, Blackpool has the highest overall rate with 30% of women smoking at the time of delivery in 2011/12, which is over twice the national average for England (13%) [36]. *Incentive culture:* Lancashire is an innovative area for breastfeeding incentive schemes. The ‘Be a Star’ www.beastar.org.uk/archives/tag/be-a-star-adverts-lancashire campaign started in Lancashire in 2008 and promotes breastfeeding amongst 16–25 year old mothers. It originated as a partnership between one of the Primary Care Trusts, Little Angels breastfeeding peer support organisation and The Hub social marketing agency. Be a Star transforms local breastfeeding mums to look like models, celebrities, singers and actresses, making breastfeeding glamorous, sexy and appealing in posters and provides breastfeeding support. Be a Star has been rolled out across 15 Primary Care Trusts in England with encouraging results. The Strategic Health Authority provided funds to three areas in the North-West (one of which is NHS Blackpool Primary Care Trust) to run incentive schemes with the aim of increasing breastfeeding duration at 6–8 weeks in 2011 by 5%. The community Star Buddies Breastfeeding Peer Supporters who are delivering the incentive scheme in Blackpool operate out of St Cuthbert’s and Palatine Children’s Centre (our co-applicant base).

Qualitative research involved purposive, theoretical and snowball sampling undertaken by five interviewers (GT, NC, HM, JMcK, SM) across the three sites to include individuals with and without experience of incentive interventions. Participant recruitment was facilitated by discussions with service managers and key workers in health, social and third sector organisations. There were three sample populations: a) pregnant women, new mothers and their partners/family members who had become parents within the last 6 months; b) providers who could either deliver or receive incentives to support women to initiate or maintain smoking cessation or breastfeeding and c) experts and decision makers either in a management/coordinator position potentially responsible for implementing incentive programmes, e.g. a policy maker at local, regional or national level or a member of a research ethics committee.

#### Data collection

A range of qualitative methods were used between November, 2012 and June, 2013, including unstructured semi-structured and structured interviews with vignettes, focus groups, interactive discussions and four open questions on a web survey [14]. Our iterative approach to data collection and analysis continually generated new research questions. For each question we discussed as a research team the most appropriate qualitative method for data collection. For example, to understand unintended consequences for specific promising interventions identified in the evidence synthesis, we used vignettes to describe the aspects we were interested in and identified participants (i.e. women, partners, providers) who potentially could have been involved in such an intervention to ascertain their views. To seek disconfirming data, we conducted more unstructured individual interviews, rather than a group discussion where there is a tendency towards consensus, i.e. FG11 which involved a focus group with health visitors from one geographical location.

Semi-structured interviews were used to explore participants’ knowledge, experiences and attitudes towards incentive provision and the potential implications and consequences of incentives. Topic guides were designed with the involvement of service users and were modified iteratively as the study progressed. Eight vignettes were developed from studies identified in the systematic reviews, which were selected either because they had statistically significant effects or involved an unusual, promising or innovative approach. Six vignettes were used with mothers/partners/family members [37]–[42] and four with professionals [34], [37], [39], [43] (Table 2). The vignettes were included in interviews and focus groups to garner participants’ perspectives of the incentive intervention. On two occasions, interviews were held in the women’s homes; the remaining interviews/focus groups were held at community or health and social care services locations (e.g. mother and baby group, health clinic) or via the telephone. Interviews/focus groups ranged from ∼15 to 100 minutes and were audio recorded and transcribed in full.

**Table 2:** Intervention vignettes.

SMOKING CESSATION STUDIES
*Gulliver et al* [38]
You and your partner/relative are invited to attend a 60 minute group with other expecting couples which is led by a psychologist at a local hospital, to discuss pregnancy and smoking.In the group, the psychologist wants to find out whether you are ready to give up smoking and if your partner/relative can help you. The group is told that giving up smoking is possible and how it would improve their own and their baby’s health. You and your partner/relative are also invited to couple counselling appointments to discuss your own experiences of smoking, and previous attempts to stop. You are told that the counselling appointments will include: working with a self-help manual (Freedom from Smoking for You and Your Baby) and thinking about the triggers for smoking. You will be asked to sign a contract for your chosen stop smoking plan. You are then invited to attend monthly appointments until your baby is three months old. At these appointments, you are asked about your smoking and have a breath test to show whether you are still smoking or not. At each visit there is a raffle which you can enter to win gifts, regardless of whether you are still smoking or not. A car seat is raffled every three months. Also, if you stay quit, and the breath test proves it, you will be given additional gifts donated by local businesses as they want to support your efforts to stay smoke free. All your travel to and from appointments will be paid for.
*Heil et al* [39] *– Version used with women/partners/other family members*
At 18 weeks pregnant, you are invited to attend a stop smoking appointment. There, you are asked to agree a quit date, give a breath test and provide a urine sample and you are also given a smoking cessation leaflet, which you discuss with staff members. If you agree to continue the service, you will have tests to assess whether you are still smoking:
- every day for the next 5 days
- then twice weekly for another 7 weeks
- once a week for the next 4 weeks
- then fortnightly up until the baby is born
After your baby is born, you will also have to provide samples:
- every week for 4 weeks
- then fortnightly for the next 8 weeks (12 weeks in total)
- at a final assessment made at 24 weeks after the baby is born
You will receive vouchers for as long as you stay quit and these increase in value each time the test confirms that you have stopped smoking (starting from £10.00 at first testing, and then increasing by £2.00 for each negative test – up to a maximum of £70.00). Any positive/missing results will reset the value of the vouchers (to £10.00), however if you then have a further 2 negative results, the value of vouchers will be restored. During each visit you will discuss your smoking status and the benefits of not smoking during pregnancy/after the birth; and at the end you will receive a pamphlet highlighting the reasons to remain non-smoking.
*Heil et al* [39] *– Version used with professionals*
At 18 weeks gestation, women are recruited to a smoking cessation intervention that involves daily, bi-weekly and then weekly contacts until the baby is born, with further weekly and fortnightly contacts up to 12 weeks postnatal (with a final contact at 24 weeks). Urine and CO_2_ tests are used to confirm smoking status on each occasion, and women are given opportunities to read/review smoking cessation information with a health worker. A voucher is given, which increases in value (maximum of £40) each time a negative smoking test is confirmed, but values are re-set if a positive test is received.
*Walsh et al* [42]
You and your partner have been invited to attend a three session smoking cession programme. The programme consists of the following:
*First session:*
- You are given 2–3 minutes of risk information advice from a doctor, and shown a 14 minute video that contains risk information, barriers to quitting and how to overcome them and stop smoking tips.
- Following the video, a 10 minute counselling session is provided by a midwife and a quit date is agreed.
- You receive a self-help manual as well as guidance on how to use it (this manual includes sections on risks, barriers, and smoking cessation).
- You and your partner are offered four packets of confectionary gum.
- Your partner is provided with a tip sheet, a contract and letter that stresses the importance of smoking cessation support.
- A sticker is placed on your medical records so that other professionals know that you are involved in the programme
*Second and third sessions (held at approx. 34–36 weeks of pregnancy):*
- On the 2nd and 3rd visits, a midwife will provide approximately 5 minutes of counselling support and a doctor will provide approximately 2 minutes of risk advice.
- Urine samples will be collected during these visits to test whether you are smoking
*Follow-up:*
- You will provide a further urine sample between 6–12 weeks after your baby has been born
- If your urine sample (provided at your second visit) is negative, your name is entered into a draw to win four donated prizes (approx. £120 each).
*Tappin et al* (CPIT study) [34]
Pregnant smokers are given a £50 voucher for attending an appointment with an NHS Smokefree Pregnancy advisor and setting a quit date. They are given an additional £50 voucher for being smoke free 4 weeks after their quit date and another £100 voucher for being smoke free after 12 weeks. If they are still smoke free towards the end of their pregnancy, they are given a further £200 voucher. Vouchers can be exchanged at many retailers.

BREASTFEEDING STUDIES
*Chamberlain et al* [37] *– Version used with women/partners/other family members*
You are offered a personal use double-electric breast-pump (worth £120–225, your brand of choice), which can be delivered to the hospital or to your home following the birth of your baby. You will have access to a breastfeeding specialist in the hospital and you will be given a number for a breastfeeding telephone support line that you can call from home.
*Chamberlain et al* [37] *– Version used with professionals*
A mother is offered a personal use double-electric breast-pump (worth £120–225, her brand of choice), which can be delivered to the hospital or to her home following the birth of her baby. Whilst the mother is breastfeeding, she will have access to a breastfeeding specialist in the hospital and given a number for a breastfeeding telephone support line that she can call from home.
*Pugh et al* [40]
Within 24 hours of having your baby you are asked whether you would like to take part in a parenting/breastfeeding programme. Once you have agreed, you are asked to complete some questionnaires and provided with breastfeeding support. You are told that you will receive:
- At 3–4 days following the birth a nurse will visit you to discuss parenting issues/provide breastfeeding support.
- At 5 days following the birth a breastfeeding specialist will telephone you to discuss breastfeeding
- At 12 days after the birth, the nurse will visit you and offer flexible ‘non-nursing’ support based on what support you need, e.g. washing dishes, doing the laundry, providing child-care.
- You are also told that further questionnaires will be sent to you for completion at 14 days and 6 weeks after the birth of your baby.
*Volpe et al* [41]
During your pregnancy, you are invited to attend three 1-hour weekly group sessions that provide education, information and support for parenting issues and breastfeeding. Each week will focus on a different topic and you will be provided with a gift at the end of each session. The focus of the sessions and the gift provided will be:
Week 1 (healthy eating) – chocolate cigar
Week 2 (safety) – electrical outlet covers
Week 3 (mothering the mother) – perfume
The sessions are run by a nurse/breastfeeding specialist – and a breastfeeding supporter will also be present to provide support and encouragement. The breastfeeding supporter will continue to visit you after your baby has been born if you continue to breastfeed.
*Cattaneo et al* [43]
The Regional Health Authority has requested local health authorities to develop local work plans and targets to increase breastfeeding rates. The rates of exclusive, predominant and complementary breastfeeding are to be measured at birth and at 16–19 weeks of age. All staff working within the health authority are told that a financial penalty will be assigned if they do not achieve their objectives and targets

GT, HM, NC and PH facilitated and recorded interactive discussions at three conferences (maternity/early years and public health) comprising academic, third sector and health professionals over the course of the study period to discuss the feasibility and acceptability of incentive provision for consumers and providers.

### Survey data

#### Setting, participants and data collection

The web survey was distributed to maternity and early years staff working in Scotland and North West England (see Appendix S1 for a full copy of the survey). Recipients were identified and contacted by health service gatekeepers to email lists for maternity services, child health and primary care via NHS Research and Development Networks, the Scottish Primary Care Research Network (SPCRN) and a private company. The emails provided an introduction to the study with a link to Survey Monkey (www.surveymonkey.com). With the exception of those reached through SPCRN, to whom one reminder was sent by each of the regional co-ordinators to their respective area email lists, no repeat emails were sent. All respondents were offered entry into a draw to win one of forty £5 retail vouchers.

#### Survey design

A shortlist of seven promising incentive strategies emerged from the BIBS study evidence syntheses [14], [15], service-user feedback and early qualitative data collection. This shortlist informed the incentive descriptions used in the survey to investigate health care professional acceptability and anticipated consequences [14]. Survey questions asked participants to respond using a 5 point Likert scale (1- strongly agree; 5- strongly disagree) to seven promising incentive strategies identified from evidence syntheses and qualitative interview data. Five were incentives to women, including a free breast pump and conditional shopping vouchers for verified proof of smoking cessation at different time points and having a smoke-free home. Two were incentives to providers: (i) payments to local health services for reaching smoking cessation in pregnancy targets and (ii) breastfeeding targets. Demographic data and experience of target behaviours were recorded. To minimise framing effects, free text questions about positive and negative consequences were included, rather than a single question on unintended consequences. The free text survey questions were: *We would like you to imagine that your local health service is going to run a scheme that provides incentives for stopping smoking in pregnancy. What do you think the consequence might be for participants and/or staff? Qi) Positive consequences? Qii) Negative consequences?* Identical questions with *breastfeeding* inserted instead of *stopping smoking in pregnancy* followed.

### Data analysis

The first stage of analysis used a Framework approach [44] to interpret the experiences and views of participants. A key strength of the framework approach is its potential to allow data to be summarised within thematic matrices and for patterns or explanations to be identified. All qualitative interview data were entered into NVivo10 software (QSR International, Burlington, MA) to facilitate data organisation, coding and retrieval. Free-text responses to open questions in the health professional survey on the perceived consequences of incentive programmes were entered onto an Excel chart and content analysis was used to triangulate the analysis of the interview data. Data cleaning was undertaken on the survey responses in that all comments not relating to the question were recorded as ‘unsure’ to calculate a response rate for each incentive consequences question. Initially, researchers (GT, NC, HM) identified key themes and categories independently by listening to and reading transcripts of the first four participant and four provider interviews. Through wider research team transcript reading and discussion, a single tree structure coding index was agreed and applied in NVivo10 to the separate site datasets, with 2–4 weekly merges of datasets [14]. The researchers undertook a detailed analysis of data with regular discussion several times a week between sites to ensure consistency and to search for disconfirming perspectives. Drafts of the findings and analysis were circulated prior to weekly meetings with feedback provided by the project lead (PH). Analysis continued until data saturation was achieved.

A second stage of more in-depth data analysis was undertaken for this investigation of the consequences of incentives. Braun & Clark’s [45] thematic analysis was undertaken which involved reading and re-reading of all the transcripts and free text responses, followed by coding, organising and mapping the data into groups and networks until saturation occurred. Initial data analysis was undertaken by GT and the findings were shared and discussed with NC, HM and PH for consensual validation of the final emergent themes.

The qualitative research was conducted or overseen by social science and/or health researchers, three of whom had been involved in incentive interventions (GT, LB, and PH). The research team included previous smokers, those with and without children, experiences of breast and formula milk feeding who held different perspectives on incentive interventions for behaviour change. Differences and potential biases were discussed in regular team meetings and noted in reflective diaries kept by the qualitative research team.

## Results

### Qualitative participants

A total of 177 participants took part in 16 focus groups, 55 face-to-face interviews and 19 telephone interviews (Table 3). This sample included 88 pregnant women/recent mothers/partners/wider family members; 53 service providers, 24 experts/decision makers and approximately 63 conference attendees participated in an audio-recorded interactive discussion. Our interview participants represented women living in disadvantaged areas recruited within and outwith health services with experience of smoking in pregnancy and choosing not to breastfeed, ethnic diversity and educational level (Table 4). Thirty women/parents had experience of an incentive intervention (22 in the CPIT trial/incentive intervention for smoking cessation; four in a NW England breastfeeding incentive scheme; four in a third sector teenage mothers’ programme). Twenty-three CPIT providers/experts participated. Two experts had been involved in a different voucher incentive programme for smoking cessation and one expert was involved in a breastfeeding incentive intervention. More detail linking the sample characteristics to ID codes and the characteristics of who took part in the interviews and focus groups are presented in Tables 5–8.

**Table 3:** Study participants.

Participants	Number interviewed	Totals and format
**Mother and baby groups:** **co-applicant**		**Participants N = 12**
Aberdeenshire	n = 6	Focus groups^a^ n = 3
Blackpool	n = 6	Face-to-face interviews n = 2
**Pregnant women and recent** **parents**		**Participants N = 88**
Pregnant women	n = 38^b^	Focus groups^a^ n = 8
Postnatal women	n = 45	Face-to-face interviews n = 39
Partners	n = 5	Telephone interviews n = 6
**Providers**		**Participants N = 53**
Midwifery	n = 11	Focus groups^a^ n = 10
Nursing	n = 1	Face-to-face interviews n = 13
Health visiting	n = 12	Telephone interviews n = 6
Doctors: paediatricians, obstetricians, GPs	n = 5	
Public health	n = 3	
Smoking cessation specialists/staff	n = 11	
Voluntary sector/children’s centrestaff	n = 2	
Pharmacists	n = 7	
Incentive scheme administrator	n = 1	
**Experts and decision makers**	n = 24	**Participants N = 24**
		Focus groups^a^ n = 4
		Face-to-face interviews n = 3
		Telephone interviews n = 7
**Public Health, Maternal and Infant Health Conferences**	n = ∼63	**Participants N = ∼63** Interactive recorded group
Range of participants per session involvingpolicy, decision-makers, experts and some practitioners		discussions at conferences n = 3

a A total of 16 focus groups were conducted. At three focus groups with women/recent parents a provider was present and three focus groups were a mixture of providers and experts. Two women attended two different focus groups; as did two experts (they are counted once only).

b Two pregnant women were involved in a follow-up postnatal interview (one of which had an older child at the time of the first interview).

**Table 4:** Summary of characteristics of women and partner participants.

	Women/Partners	Not recorded
Ethnicity	78 (88.6%) White	1 (1.2%)
	9 (10.2%) BME	
Marital Status	68 (77.3%) Married	2 (2.3%)
	18 (20.4%) Divorced/Single	
Employment Status	43 (48.9%) Employed	5 (5.7%)
	40 (45.4%) Unemployed	
Smoking Status	26 (29.5%) Never smoked	1 (1.2%)
	24 (27.3%) Currently smoking	
	37 (42.0%) Previously quit	
Previous Infant Feeding Behaviours (N = 58)*	51 (87.9%) Previous experience of breastfeeding	3 (5.2%)
	4 (6.9%) Formula only	
Current Infant Feeding Intentions (N = 18)*	11 (61.1%) Planned to breastfeed	
	4 (22.2%) Planned to mixed feed	
	3 (16.7%) Planned to formula feed	

*Data collected on Lancashire/Aberdeen women only.

**Table 5:** Interviews - mothers/partners/other family members.

Code^1^	Parent status^2^	Age	Marital Status^3^	Ethnicity^4^	Education^5^	Employed (yes/no)	Smoking Status^6^	Liveswith smoker(yes/no)	Currently pregnant -infantfeeding intentions^7^	PreviousInfantFeeding Experiences^8^	Experience of Incentives^9^
1	Mother*	26	1	1	3	Yes	5	No	1	1	No
2	Mother	25	1	1	1	Yes	2	No	N/A	2	SC
3	Mother	38	1	1	3	Yes	5	No	N/A	1	No
T4	Mother	30	1	1	2	Yes	4	No	N/A	1	BF
5 and T6^10^	Woman	31	1	1	2	Yes	4	No	1	1	BF
7	Woman	29	1	1	1	Yes	1	No	1	N/A	No
8	Woman	35	1	2	2	No	2	No	1	N/A	No
T9	Mother	31	1	1	1	Yes	2	Yes	N/A	1	BF
T 10 and T11^10^	Mother *	29	1	1	3	No	2	Yes	1	1	BF
12	Mother*	31	1	2	3	Yes	4	No	2	1	No
13	Mother*	22	1	1	3	No	5	Yes	2	1	No
14	Mother*	26	1	1	3	No	4	Yes	1	1	SC
T15	Woman	19	1	2	3	Yes	1	No	1	N/A	No
16	Mother	39	2	1	3	No	1	No	N/A	1	No
17	Mother	53	2	1	1	No	1	No	N/A	1	No
18	Mother*	36	1	2	1	Yes	1	No	1	1	No
19	Father	38	1	1	3	Yes	1	Yes	N/A	1	No
20	Father	34	1	1	3	No	5	Yes	N/A	1	No
21	Father	48	2	1	3	No	5	No	N/A	1	No
22	Father	44	1	1	3	No	5	No	N/A	2	No
23	Man	23	1	1	3	No	5	Yes	2	N/A	No
24	Mother*	25	1	1	4	No	5	No	3	2	No
25	Mother	51	1	1	3	No	5	Yes	N/A	N/R	No
26	Woman	18–24	1	1	5	No	5	N/R	N/R	N/R	SC
27	Woman	18–24	2	1	5	No	2	N/R	N/R	N/R	SC
28	Mother*	25–35	1	1	5	No	2	N/R	N/R	N/R	SC
29	Mother*	25–35	1	1	5	N/R	5	N/R	N/R	N/R	SC
30	Woman	Under 18	1	1	5	No	2	N/R	N/R	N/R	SC
31	Mother*	25–25	1	1	5	No	2	N/R	N/R	N/R	SC
32	Woman	18–24	N/R	1	5	N/R	5	N/R	N/R	N/R	SC
33	Woman	25–35	2	1	5	No	2	N/R	N/R	N/R	SC
34	Mother*	18–24	2	1	5	No	2	N/R	N/R	N/R	SC
35	Woman	25–35	1	1	5	No	2	N/R	N/R	N/R	SC
36	Mother*	25–25	1	1	5	Yes	5	N/R	N/R	N/R	SC
37	Mother*	25–35	1	1	5	Yes	2	N/R	N/R	N/R	SC
38	Woman	18–24	2	1	5	Yes	5	N/R	N/R	N/R	SC
39	Mother*	36+	2	1	5	No	5	N/R	N/R	N/R	SC
40	Woman	18–24	2	1	5	No	2	N/R	N/R	N/R	SC
41	Woman	18–24	1	1	5	No	5	N/R	N/R	N/R	SC
42	Woman	18–24	2	1	5	No	5	N/R	N/R	N/R	SC
43	Woman	18–24	2	1	5	No	5	N/R	N/R	N/R	SC
44	Woman	25–35	1	1	5	Yes	2	N/R	N/R	N/R	SC
45	Mother*	36+	1	1	5	N/R	2	N/R	N/R	N/R	SC

N/A = not applicable; N/R = not recorded.

1 T relates to a telephone interview; no letter refers to a face to face interview.

2 Mother/father relates to those who have older children (who may/may not be currently pregnant); mother* relates to women who have older children and who are pregnant; Woman/man relates to those who are pregnant/expecting first child.

3 1 - Participant married/living together/in a relationship; 2– Single/divorced.

4 1 - White; 2 - Black or Minority Ethnic classifications (BME).

5 1- Degree level qualification; 2 - A level or equivalent; 3 - GCSE/NVQ or equivalent; 4 - No formal qualifications; 5- Not recorded.

6 1 – Never smoked; 2 – Quit during pregnancy; 3 – Cut down during pregnancy; 4 – Quit prior to pregnancy; 5 – Currently smoking.

7 Code relates to women who are currently pregnant: 1 – Plan to breastfed; 2 – Plan to mixed feed; 3 – Plan to formula feed.

8 Code relates to families with older children/interviewed in post-natal period: 1 – Previous experience of breastfeeding; 2 – Never breastfed.

9 SC – experience of smoking cessation incentive intervention; BF – experience of breastfeeding incentive intervention; ‘Other’ relates to those involved in Barnardo’s Early Years Early Action Fund; http://www.barnardos.org.uk/media_centre/press_releases.htm?ref=81644).

10 Mothers participated in an ante-natal and post-natal interview.

**Table 6:** Focus groups – mothers.

Code^1^	Parent status^2^	Age	Marital Status^3^	Ethnicity^4^	Education^5^	Employed (yes/no)	Smoking Status^6^	Lives with smoker (yes/no)	Currently pregnant – infant feedingintentions^7^	Previous'InfantFeeding Experiences^8^	Experience of Incentives^9^
FG1	Mother	28	1	1	1	Yes	4	No	N/A	1	No
FG1	Mother	38	1	2	1	Yes	4	No	N/A	1	No
FG1	Mother	33	1	1	1	Yes	1	Yes	N/A	1	No
FG1	Mother	26	1	1	1	Yes	4	No	N/A	1	No
FG1	Mother	26	1	1	1	Yes	1	No	N/A	1	No
FG1	Mother	21	1	1	3	No	2	Yes	N/A	1	No
FG1	Mother	39	1	1	1	Yes	4	No	N/A	1	No
FG1	Mother	34	1	1	1	Yes	1	No	NA	1	No
FG1	Mother	36	1	1	3	Yes	1	No	N/A	1	No
FG2	Mother	62	1	1	3	No	1	No	N/A	1	No
FG2	Mother*	26	1	1	3	Yes	1	No	1	1	No
FG2	Mother	28	1	1	3	Yes	1	No	N/A	1	No
FG2	Mother*	27	1	1	3	Yes	4	No	1	1	No
FG3	Woman	17	2	1	4	No	5	Yes	3	N/A	No
FG3	Woman	17	2	1	4	No	1	Yes	2	N/A	No
FG3	Mother	19	1	1	3	No	4	Yes	N/A	1	No
FG3	Mother	18	1	1	5	Yes	1	N/R	N/A	1	No
FG3	Mother	17	1	1	2	No	5	Yes	N/A	1	No
FG4	Mother	21	1	1	3	No	5	Yes	N/A	2	No
FG4	Mother	40	1	1	3	Yes	1	No	N/A	1	No
FG4	Mother*	30	1	1	2	No	1	No	1	1	No
FG4 & FG5**	Mother*	22	2	1	3	Yes	1	No	3	1	No
FG4 & FG5	Mother	32	1	1	3	No	5	No	N/A	1	No
FG6	Mother	34	2	1	3	Yes	3	No	N/A	1	No
FG6	Mother	30	1	1	5	Yes	4	No	N/A	1	No
FG6	Mother	34	1	1	1	Yes	4	No	N/A	1	No
FG6	Mother	28	1	1	3	Yes	2	No	N/A	1	No
FG7**	Mother	36	1	1	3	Yes	4	No	N/A	1	No
FG7	Mother	28	1	2	1	Yes	1	No	N/A	1	No
FG7	Mother	35	1	1	1	Yes	1	No	N/A	1	No
FG7	Mother	36	1	2	1	Yes	1	No	N/A	1	No
FG7	Mother	28	1	1	1	No	4	No	N/A	1	No
FG7	Mother	41	1	2	3	Yes	3	Yes	N/A	1	Other
FG7	Mother	36	1	1	1	Yes	1	No	N/A	1	No
FG7	Mother	29	1	1	1	Yes	1	No	N/A	1	No
FG7	Mother	36	1	1	2	Yes	4	No	N/A	1	No
FG7	Mother	33	1	2	5	N/R	1	No	N/A	1	No
FG7	Mother	30	1	1	1	Yes	1	No	N/A	1	No
FG7	Mother	32	2	1	3	No	5	No	N/A	1	No
FG7	Mother	N/R	N/R	N/R	5	N/R	N/R	N/R	N/R	N/R	N/R
FG8**	Mother*	19	2	1	2	No	3	No	2	1	Other
FG8	Mother	20	1	1	3	No	3	Yes	N/A	1	Other
FG8	Mother	20	1	1	2	No	5	No	N/A	N/R	No
FG8	Mother	19	2	1	3	No	3	No	N/A	1	No
FG8	Mother	21	1	1	3	Yes	1	No	N/A	2	Other

N/A = not applicable; N/R = not recorded.

1 T relates to a telephone interview; no letter refers to a face to face interview.

2 Mother relates to those who have older children (who may/may not be currently pregnant); mother* relates to women who have older children and who are pregnant; Woman relates to those who are pregnant/expecting first child.

3 1 – Participant married/living together/in a relationship; 2 – Single/divorced.

4 1 - White; 2 - Black or Minority Ethnic classifications (BME).

5 1- Degree level qualification; 2 - A level or equivalent; 3 - GCSE/NVQ or equivalent; 4 - No formal qualifications; 5- Not recorded.

6 1 – Never smoked; 2 – Quit during pregnancy; 3 – Cut down during pregnancy; 4 – Quit prior to pregnancy; 5 – Currently smoking.

7 Code relates to women who are currently pregnant: 1 – Plan to breastfed; 2 – Plan to mixed feed; 3 – Plan to formula feed.

8 Code relates to families with older children/interviewed in post-natal period: 1 – Previous experience of breastfeeding; 2 – Never breastfed.

9 ‘Other’ relates to those involved in Barnardo’s Early Years Early Action Fund; http://www.barnardos.org.uk/media_centre/press_releases.htm?ref=81644).

**Providers also took part in FG5, FG7 & FG8.

**Table 7:** Interviews – providers/experts^1^.

Participant Code^2^	Profession	Provider/Expert
T46	Consultant Obstetrician	Provider
T47	Research Manager Voluntary Sector	Expert
T48	Public Health Consultant	Expert
T49	Health Visitor	Provider
T50	Health Visitor	Provider
T51	Lead Health Trainer – Smoking Cessation	Expert
52	Specialist Midwife (Substance misuse)	Provider
53	Hospital Midwife	Provider
T54	Senior Clinical Lecturer/Ethics CommitteeMember	Expert
T55	Consultant Obstetrician	Provider
T56	Tobacco Trainer - Tobacco Control Team	Provider
T57	Stop Smoking Service Manager	Expert
T58	Smoking Awareness Co-Ordinator	Expert
T59	Midwife	Provider
T60	Infant Feeding Co-Ordinator	Expert
T61	Smoking Cessation Advisor	Provider
62	Ethics Committee Member	Expert
T63	General Practitioner	Provider
T64	Paediatrician (neo-natal)	Provider
T65	Paediatrician (general and respiratory)	Provider
66	Health Improvement Senior Officer	Expert
67	Helpline Manager	Expert
68	Midwife	Provider
69	Community Midwife	Provider
70	Research Nurse	Provider
71	Senior Midwife	Provider
T72	Smoking Cessation Advisor	Provider
T73	Smoking Cessation Advisor	Provider
T74	Incentive Scheme Administrator	Provider

1 Nine CPIT providers/experts took part in an interview; two experts were involved in a voucher incentive intervention for smoking cessation.

2 T relates to a telephone interview; no letter refers to a face to face interview.

**Table 8:** Focus groups & interactive discussions – providers/experts^1^.

Participant Code	Profession	Provider/Expert
FG5	Peer Supporter*	Provider
FG7	Health Visitor*	Provider
FG7	Health Visitor*	Provider
FG7	Student Nurse/Health Visiting*	Provider
FG8	Liaison Worker for Young Mums - Voluntary Sector*	Provider
FG9	Senior Public Health Coordinator	Expert
FG9	Assistant Director of Nursing and Families	Expert
FG9	Infant Feeding Consultant	Expert
FG9	Infant Feeding Coordinator^2^	Expert
FG9	Baby Friendly Coordinator	Expert
FG10	Public Health Specialist	Provider
FG10	Parentcraft and Infant Feeding Coordinator^3^	Expert
FG10	Children’s Centre Development Officer	Expert
FG10	Infant Feeding Coordinator	Expert
FG10	Breastfeeding Peer Support Branch Manager	Expert
FG10	Breastfeeding Peer Support Operations Manager	Expert
FG10	Breastfeeding Peer Support Coordinator	Expert
FG10	Public Health Coordinator	Expert
FG10	Health Coordinator Children’s Centres	Expert
FG11	Health Visitor	Provider
FG11	Health Visitor	Provider
FG11	Health Visitor	Provider
FG11	Health Visitor	Provider
FG11	Health Visitor	Provider
FG11	Health Visitor	Provider
FG11	Health Visitor	Provider
FG12	Public Health Practitioner	Provider
FG12	Health Education Practitioner	Provider
FG13	Community Midwife	Provider
FG13	Community Midwife	Provider
FG13	Community Midwife	Provider
FG13	Community Midwife	Provider
FG13	Community Midwife	Provider
FG13	Community Midwife Coordinator	Expert
FG14	Community Pharmacist	Provider
FG14	Community Pharmacist	Provider
FG14	Community Pharmacist	Provider
FG14	Community Pharmacist	Provider
FG14	Community Pharmacist	Provider
FG14	Community Pharmacist	Provider
FG14	Community Pharmacist	Provider
FG15	Smoking Cessation Advisor	Provider
FG15	Smoking Cessation Advisor	Provider
FG15	Smoking Cessation Advisor	Provider
FG15	Smoking Cessation Advisor	Provider
FG15	Smoking Cessation Advisor	Provider
FG16	Helpline Staff	Provider
FG16	Helpline Staff	Provider
IA1^4^	Infant & Nutrition Conference 2011 (n = 30+)	Providers & Experts
IA2^4^	Infant & Nutrition Conference 2013 (n = 15)	Providers & Experts
IA3^4^	Public Health Conference 2012 (n = 18)	Providers & Experts

1 14 CPIT providers took part in focus groups; one expert was involved in NW England breastfeeding incentive intervention.

2 Participant also took part in FG10.

3 Participant also took part in FG12.

* Participants took part in focus groups with women.

4 Interactive discussions that involved a mixture of practitioners and experts, a number of who had been involved in smoking cessation/breastfeeding incentive interventions.

### Survey participants

A total of 497 health and early years professionals responded to the survey. The characteristics of the respondents are reported in Table 9. The response rates to the free text questions are detailed in Table 10. This table indicates that survey participants were more likely to record positive rather than negative consequences for both target behaviours. Most positive comments concerned the expected health benefits associated with not smoking and breastfeeding, and the intended consequences of incentive delivery of smoking quit rates and increased breastfeeding.

**Table 9:** Characteristics of the maternity and early years health professional sample (n = 497).

Variable	Classes	Sample (%)
**Sex**	Male	64 (12.9)
	Female	411 (82.7)
	Missing	22 (4.4)
**Age**	18–34	91 (18.3)
	35–44	114 (22.9)
	45–54	182 (36.6)
	55>	85 (17.1)
	Missing	25 (5.0)
**Ethnicity**	White	444 (89.3)
	BME/prefer not to say	53 (10.7)
	White British	339 (68.2)
	White Irish	7 (1.4)
	White Other	1 (0.2)
	Mixed W/B Caribbean	1 (0.2)
	Mixed Other	1 (0.2)
	Asian Indian	10 (2.1)
	Asian Pakistani	2 (0.4)
	Asian Chinese	1 (0.2)
	Black African	2 (0.4)
	Refused	35 (7.0)
**Smoking status**	Never smoked	370 (74.5)
	Current smoker, tried to stop smoking	17 (3.4)
	Current smoker, not tried to stop smoking	1 (0.2)
	Ex-smoker	101 (20.3)
	Declined to answer	8 (1.6)
**Any children**	Yes	401 (80.7)
	No	96 (19.3)
**Breastfeeding**	Any children breastfed	387 (77.9)
	No children breastfed	110 (22.1)
**Job**	General Practitioner	132 (26.6)
	Health visitor	47 (9.5)
	Manager	20 (4.0)
	Midwife	121 (24.4)
	Obstetrician	12 (2.4)
	Maternity staff	29 (5.8)
	Paediatrician	12 (2.4)
	Other nurse	41 (8.3)
	Public health staff	32 (6.4)
	AHP	18 (3.6)
	Support role	8 (1.6)
	Researcher	4 (0.8)
	Missing	21 (4.2)
**Survey region**	England	60 (12.1)
	Scotland	437 (87.9)

**Table 10:** Response rates to free text questions in the professional survey (n = 497).

	Positive consequencesof incentivesto participantsand/or staff(smokingcessation)	Negativeconsequences of incentives toparticipants and/orstaff(smoking cessation)	Positive consequencesof incentives to participantsand/orstaff (breastfeeding)	Negative consequences of incentives to participants and/or staff (breastfeeding)
Provided comments N (%)	377 (75.9%)	372 (74.9%)	358 (72.1%)	338 (68.0%)
No data entered. N (%)	93 (18.7%)	102 (20.5%)	110 (22.1%)	121(24.3%)
Stated “no consequences” or “unsure” N (%)	27 (5.4%)	23 (4.6%)	29 (5.8%)	38 (7.6%)

The four emergent themes that integrated positive and negative unintended consequences are: addressing or creating inequalities; enhancing or diminishing motivation and wellbeing; relationships with others; and impact on health systems and resources. These are summarised in Table 11. In the following sections, each theme is described and illustrated with quotes from participants. Participant quotes have been assigned a code, for example (FG5, I, mother), which describes whether the participant took part in a focus group (FG), interactive discussion (IA), survey (S), telephone interview (T), or face-to-face interview (no code), and gives the participant identification number. The code also indicates whether or not the participant had been involved in an incentive programme (I versus no letter) and describes participant characteristics: professional background, whether the mother was pregnant for the first time (pregnant mother), a mother (who may or may not have been pregnant again) or a wider family member. Where appropriate, the findings have been integrated across the different participant groups. Where distinctions between participant groups are relevant, the term ‘consumer’ has been used for ‘women/partners/family member’ comments, and ‘professional’ relates to comments from providers/experts/conference attendees.

**Table 11:** Key emergent themes for unintended consequences of incentives.

Addressing or Creating Inequalities	
*Positive*	*Negative*
- Encouraging/maintaining access to health	- Most disadvantaged less likely to access
care	- Unfairness of incentives
- Provides disadvantaged families	- Marginalisation/divisive
with additional income/items not able to	- Post-code lottery of care
Afford	- Withdrawal from health services
**Enhancing or Diminishing Intrinsic Motivation and Wellbeing**	
*Positive*	*Negative*
- Tipping point	- Gaming & cheating/fallibility of proof
- Reward and recognition	- Limited efficacy once incentive withdrawn
- Increased motivation and job satisfaction	- Reduction in staff motivation
- Rare opportunity for autonomy and choice	
**Relationships with Others**	
*Positive*	*Negative*
- Shared aim	- Impaired provider-women interactions
- Positive provider-women relationships	- Negative social, emotional and health impact of
- Encourage others to adopt health	non-incentivized others within personal networks
behaviours	- Nanny state, reduced autonomy
- Wider community benefits	- Pressure, guilt and perceptions of failure
**Impact on Health Systems and Resources**	
*Positive*	*Negative*
- Endorsement and focus by health services	- Negative publicity for health services
- Cost savings of improved health	- Waste of health service resources/opportunity
- Specialist training/targeted provision	costs
	- Expect ‘payment’ for other health behaviours
	- Increased workload for staff

### Addressing or Creating Inequalities

Financial incentives for smoking cessation and breastfeeding when discussed in relation to the Tappin et al. [34] and Heil et al. [39] intervention vignettes were considered to have ‘*appeal*’ particularly amongst teenage mothers and/or families ‘*where money is an issue*’ to ‘*attract*’ or ‘*persuade*’ them to engage in health services and for a ‘*meaningful conversation*’ to enable them to make ‘*informed choices*’ regarding their health behaviours:

*I think if you were young, or if you were on your own and you might feel a big judged at times, or a bit….you know, why*’*s this person coming to look at me again, you know and I think the incentive scheme can only kind of help that really and make it a nicer experience* (T9, I, mother).

Professionals perceived consumer incentives to be a ‘*foot in the door*’, leading to a ‘*greater uptake of services*’ particularly for ‘*those in need*’, leading to ‘*improved health outcomes and opportunities to engage in other health advice*’. However, the capacity to increase health inequalities due to marginalised families and those with very chaotic lifestyles being less likely to be aware of, and engage with, incentive provision was a concern amongst a number of the professionals:

*It*’*s those that understand the system that benefit most and they*’*re the ones that least need it. So if there was an incentive scheme you can be pretty sure that everyone earning between £25,000 and £70,000 a year will be taking advantage of that incentive scheme whereas those who are on £8,000 a year won*’*t even know about the incentive scheme* (FG12, providers & expert).

Concerns were raised by participants that incentive withdrawal consequent on continued smoking or relapse, or on reduced or discontinued breastfeeding could lead to women being less likely to ‘*report problems*’ or ‘*non-engagement*’ due to professionals ‘*going on at me to give up* [smoking]’ or continue breastfeeding.

Incentives for smoking cessation were considered by many of the participants to be ‘*unfair*’, for example, by ‘*rewarding*’ smokers who are doing ‘*something they know to be detrimental to health*’ or penalising those who ‘*are doing the right thing*’. Some perceived this as counter to parenting and education practices in terms of ‘*positive reinforcements*’ for good, rather than negative behaviour:

*If you are a pregnant mum and not smoking, you should be incentivised because you are being the role* [model] *so that might give the others mums who smoke motivation to stop, knowing what they could get so you are rewarding the good behaviour* (FG2, mothers).

Many professionals also raised concerns when discussing the Cattaneo et al intervention vignette [43] about how incentives to providers associated with meeting targets for smoking cessation and breastfeeding would be ‘*inequitable*’, ‘*unrealistic*’ and ‘*unfair*’ when women’s decisions and choices were considered largely outside of their control, particularly for those working in areas of high deprivation where smoking and formula feeding are more prevalent.

Other participants felt that incentives could ‘*stigmatise*’ and create ‘*polarisation*’ and ‘*discrimination*’ between different groups of women (e.g. those who breastfeed and those who formula feed), and even ‘*resentment*’ if the incentive was targeted towards a particular, ‘*undeserving*’ population: ‘*well that*’*s my money, going to my next door neighbour, they don*’*t have a job*’.

For geographically targeted incentives, some participants expressed concerns towards a ‘*postcode lottery*’ of care and believed ‘*equity*’ to be important as everyone needs support. However, universal incentives could ‘*benefit those who already had enough*’. Some professionals believed that targeted provision could help address embedded ‘*social norms*’ associated with the target behaviours. Many participants considered how incentives had the potential to reduce inequalities through providing access to items that they could not afford (e.g. breast pump, nursing bras), and financial support for those who are ‘*struggling for money*’ to buy essentials such as ‘*food*’, ‘*things for the baby*’ or for a ‘*healthier lifestyle*’:

*Obviously it is an expensive thing, having a baby and I think that people who maybe need the income more, it would help massively. If I*’*d been in that boat it would have helped massively* (44, I, pregnant woman).

### Enhancing or Diminishing Intrinsic Motivation and Wellbeing

Numerous participants, in discussion of intervention vignettes such as Tappin et al. [34] and Heil et al. [39] believed that incentives for smoking cessation and breastfeeding could operate as a ‘*tipping point*’ to ‘*encourage*’ and provide an ‘*extra boost*’ for women to adopt healthy behaviours; with the ongoing delivery of incentives providing ‘*something to look forward to*’ to ‘*push you* [woman] *a bit further*’. Incentives could provide recognition of providers and consumers’ achievements promoting ‘*well-being*’, ‘*self-esteem*’ for women, and for providers ‘*job satisfaction if the rates improve*’.

Some consumers and providers who had been involved in incentive schemes for smoking cessation and breastfeeding referred to how women felt ‘*privileged*’, ‘*valued*’ and more ‘*confident*’:

*I felt quite privileged they picked me for any group* [research study incentive group] […] *I felt quite happy that they had actually considered putting me in one of the groups because I never thought I was going to get into any of the groups to start with* (43, I, pregnant woman).

A few professionals reflected that incentives would provide vulnerable individuals with one of the first opportunities to receive a reward and acknowledgement for an achievement. Unrestrictive incentives like shopping vouchers could provide the most disadvantaged families with a rare opportunity for autonomy to ‘*make decisions about what they ought to be spending the additional money on*’, such as providing ‘*treats*’ for themselves and their families.

Other professionals when discussing the Cattaneo et al vignette [43] considered how provider incentives could diminish health professional’s intrinsic motivation to support the target behaviours:

*Then they* [health professionals] *are not actually being motivated by increasing people to breastfeed, they are going to be motivated by the fear of the humiliation if they don*’*t get there* (IA2, providers & experts).

A number of the participants believed that incentives for smoking cessation could diminish women’s personal motivation by discouraging quitting prior to enrolling in the programme, thereby reducing ‘*health choices to a financial transaction*’, or even incentivise ‘*people to get pregnant*’. While some consumers considered this unlikely; ‘*why damage your body if you don*’*t actually already do it*’, incentives of higher value were generally believed to be associated with a greater likelihood of ‘gaming and cheating’ ‘*but £750, like I say I would I start smoking for that*’. Relapse after the incentive had been withdrawn, due to ‘*people stopping for the wrong reasons*’ and re-access ‘*get the voucher, spend it, then start again*’ were highlighted by many.

Participants also raised apprehensions with regard to vouchers that had a ‘currency’ value and could be exchanged for inappropriate items, e.g. ‘*cigarettes*’, ‘*formula milk*’ ‘*illicit drugs*’ or ‘*alcohol*’. Restrictions on voucher use, such as within the CPIT trial where general shopping vouchers could not be used for cigarettes or alcohol, or behaviour related incentives (i.e. a breast pump discussed in the context of the Chamberlain et al [37] intervention vignette) or small low value personal gifts were felt to be less open to abuse:

*You can imagine they are probably open to some sort of manipulation especially if the rewards are financial rather than the recognition of a mile stone, a badge, a picture frame* (T6, I, mother).

Other ‘gaming and cheating’ concerns were raised by participants with regard to how provider incentives may lead to staff ‘*skewing*’ their records, or women taking ‘*advantage*’ of the schemes, through making ‘*fraudulent claims*’ and ‘*lying*’ to ensure eligibility. These anxieties were magnified due to the perceived fallibility of ‘proof’; ‘*no real way of knowing if women are actually continuing to breastfeed*’. For smoking, there was some concern expressed that women may learn how to cheat when given Carbon Monoxide (CO) breath tests to confirm smoking status. CO can only capture recent smoking (in the past 12 hours) and there was some speculation that women might be able to abstain just for that period to obtain the incentive. However, this issue was not evident within the CPIT trial data from service providers:

*So far the people that I have engaged with that have signed up to the service to me, bar one, I feel have been one hundred percent genuine* (T72, I, smoking cessation advisor).

Many participants considered that verification of behaviour change was crucial to prevent the schemes coming into ‘*disrepute*’.

### Relationships with Others

When discussing the Walsh et al. [42] and Cattaneo et al. [43] intervention vignettes, some participants considered how incentives to providers to meet targets for smoking cessation in pregnancy and breastfeeding could contribute to a ‘*shared aim*’ across different individuals and services and getting ‘*everybody on board in some shape or part*’. Incentives to consumers could also operate as ‘*enablers*’ for ongoing contact with services:

*Sometimes, when you are feeling like rubbish and your house is a mess and you think “oh I can*’*t be bothered with somebody else coming round now”, you*’*ve got the health visitor and you*’*ve got you know all the other…. and you think “oh another person coming round to look at me”, but I think the incentives definitely can well …I will get a present from this one* (T9, I, mother).

Regular women–provider contact, such as depicted within the Heil et al. [39] intervention vignette and the opportunity to demonstrate ‘*value*’ to women through incentive delivery was believed to help create a ‘*positive effect*’ on the women–provider relationship, assist women-centred care and make it ‘*easier for staff*’ to encourage women to adopt healthy behaviours.

From a counter perspective, women ‘*being paid to do something that they should do without question for the health of the baby*’ could cause resentment amongst providers. A few professionals voiced concerns of feeling ‘*embarrassed*’ in ‘*selling*’ incentives to women. Many consumers as well as professionals in response to the Cattaneo et al intervention vignette [43] reported that provider incentives could lead to women ‘*feeling bombarded*’, ‘*bullied*’ or ‘*inappropriately handled*’ in attempts to ‘*manipulate people into a particular behaviour*’; which in turn could exacerbate unhealthy behaviours, i.e. women ‘*smoking even more*’ or being less inclined to breastfeed. Other concerns were how providers may incorporate ‘*bias and opinions rather than research and fact*’ and provider–women interactions relegated to a ‘*tick-box*’ exercise; with efforts focused on targets ‘*rather than healthcare*’ and neglecting non-target areas: ‘*bottle-feeding women having even less support*’.

Some participants considered how incentives delivery could negatively impact on the ‘*therapeutic relationship*’ creating ‘*distrust*’ between women and providers, with women left feeling ‘*judged and alienated from their health care givers*’:

*You*’*ve got to think about how these mums feel when they don*’*t want to breastfeed at all, then you*’*re bombarding them with it, and they won*’*t have any relationship and you*’*ve lost every other good thing you could do with them* (FG11, health visitors).

A few participants reported that women’s involvement in incentive schemes could create ‘*social connectivity*’ through being ‘*part of something*’ and knowing that their involvement ‘c*ould help other people*’. However, failure or relapse could create additional strain on women through fears of ‘*letting her* [provider] *down*’. Withdrawal or non-eligibility of incentive schemes for women who were unable to breastfeed or quit smoking was considered by many participants to ‘*penalise those who have tried*’ creating ‘*stress*’, ‘*pressure*’ and ‘*blame*’, leading to ‘*reduced self-esteem*’, ‘*depression*’ ‘*guilt*’ and feelings of ‘*failure’* and *‘inadequacy’*:

*Breastfeeding is not always a personal choice whether you can or not, whereas smoking you are personally responsible, whereas breastfeeding no matter how hard you try you might not be able to do it* (17, grandmother).

A number of professionals, in reflection of the Cattaneo et al intervention vignette [43] believed that provider incentives would create stress through staff having to *‘confront’* and *‘challenge’* those whose *‘claims are disproved’*. Some professionals also expressed that the pressure of target attainment could ‘*demoralise’* staff, leading to a situation where they ‘*disengage and don’t deliver effectively’* or even result in an ‘*increase in sickness levels’*.

Many participants considered incentive provision to be *‘patronising’* and to ‘*infantilise women’* through re-enforcing a *‘paternalistic’, ‘nanny state’* ethos. The potential for creating new social or health care norms could potentially diminish individual responsibility for making healthy choices and shift the balance ‘*from being a responsible mother to “the NHS will sort me out”’*:

*We’re health, you know. It’s your own health, take some responsibility. I think we’re going right down the wrong route; I think that, you know, when we’re enticing people with money and gifts just to do what’s right for their health’* (53, midwife).

Signing up to an incentive scheme was believed to have wider benefits for healthy behaviours ‘*cascading’* to staff, family members and other members of the women’s personal networks; creating ‘*health improvement for all the family*’ and ‘*healthy competition*’ in achieving behaviour change. Consumers and professionals through discussion of the Gulliver et al. [38] and Heil et al. [39] intervention vignettes for smoking cessation also felt that while it was harder for partners to stop smoking ‘*as a woman has a reason to*’; the potential to engage them could help to share the responsibility for health, rather than the sole onus being placed on the woman:

*Both of them are in it together then, they*’*re not feeling as if one is responsible for this if the baby is compromised, you know, I would hope even if they didn*’*t stop smoking, which I hope that they would, but if the baby was compromised they couldn*’*t blame one or other. It*’*s meeting the point that both of them are responsible for this baby* (T59, midwife).

Participants also expressed ‘*vested interests*’ in supporting incentive schemes to prevent passive smoke exposure *‘being surrounded by people puffing away on fags*’; encouraging women to ‘*set a good example*’, ‘*raise community awareness*’ and create ‘*positive cultural change*’ by normalising not smoking and breastfeeding:

*If breastfeeding becomes more, people start thinking of breastfeeding once again as the norm then it will be easier for other people to do it, until it gets to such a point where it will be considered unusual to be going onto formula feed a baby straight after birth* (T6, I, mother).

Conversely, some participants felt that the adoption of positive health behaviours could lead to social isolation for women ‘*if all their family and friends are smokers*’. The women’s efforts may be thwarted by ‘*unsupportive partners and family members*’ and have adverse consequences for personal network relationships with ‘*everyone smoking around me*’. The health benefits of quitting smoking in pregnancy were seen by some as subsequently ‘*erased*’ if the baby still resides within a ‘*smoking household*’.

A few professionals raised concerns about ‘*domestic abuse*’ in terms of how women may be pressurised by partners to sign up when they are ‘*maybe not really 100% wanting to*’ or for ‘*people taking those vouchers off those women as they walk out the door*’.

### Impact on Health Systems and Resources

Investment in incentive programmes, despite the ‘*hard strapped*’ situation of the UK NHS was perceived by many of the participants to be an important endorsement of the ‘*seriousness*’ of not smoking and breastfeeding and a positive ‘*pro-active*’ rather than reactive stance to health promotion. A number of the participants considered how incentives were not ‘*a lot of money*’ compared to the long-term savings on health-related conditions associated with smoking and not breastfeeding; although some felt that evidence was needed prior to wide-scale rollout:

*I think it*’*s fantastic if it does work, because really if four hundred pounds during pregnancy does work fantastic to the cost of the NHS because you can have so much less extra scans during the pregnancy, complications during the pregnancy, less babies born premature, less still birth, less cot death, it*’*s going to cost the NHS thousands and thousands and thousands of pounds, millions really* (FG15, I, smoking cessation advisors).

Cost savings due to reduced workloads for health service staff and how a ‘*healthier workforce*’, particularly in relation to smoking cessation, would result in employers having lower absenteeism rates were raised. Some professionals also stated how organisation incentives to meet targets could provide invaluable opportunities for needs-led service development; ‘*to buy something for our service*’.

From a more negative perspective, many participants believed that incentive programmes could create a ‘*bad image*’ and ‘*negative publicity*’ as a ‘*waste*’ of tax payers’ money and health service resources. In the current adverse economic climate, participants considered incentives to be the ‘*wrong use of money*’ when the health service was faced with ‘*cut backs affecting visitation times*’ and hospitals ‘*trying to clear debts*’ as well as the opportunity costs for resourcing other services:

*If they* [general public] *thought they were actually getting money, just because there is so many cut backs, they may be seeing it that they*’*ve got an elderly relative with Alzheimer*’*s or something like that or nursing home fees* (66, I, health improvement senior officer).

Others expressed how the increased expectations of payments for ‘other’ health behaviours, such as ‘*obesity*’, ‘*healthy eating*’, ‘*alcoholics*’ or ‘*drug addictions*’ could be a consequence:

*Because if you start, obviously with the incentives with the breastfeeding, and they are smoking, well where do you draw the line* (T11, I, mother).

The Cattaneo et al vignette [43] stimulated views from professionals that provider incentives were important to focus efforts on these behaviours and make sure ‘*staff are more highly skilled*’. CPIT providers reported how the initial ‘*chaos*’ and ‘*resistance*’ experienced when the incentive scheme was introduced ‘*settled down overtime*’. However, many professionals raised concerns about the associated costs of appointing committed, dedicated and skilled staff to deliver incentive programmes, and the potential implications of training, paperwork, administration, organisation, delivery and ‘*policing*’ of incentive delivery on ‘*overstretched staff*’ who were faced with ‘*competing priorities*’:

*More staff input is a costly affair and staff are already under stress due to lack of time and resources so extra people would have to be recruited to implement these incentives and not just be added on to job descriptions* (S164, midwife).

## Discussion

To our knowledge, this is the first study to investigate the experienced and anticipated unintended consequences of incentive provision to either women or service providers for smoking cessation in pregnancy and breastfeeding. Findings highlight controversial and oppositional views towards financial incentives with ethical, political, cultural, social and psychological implications. We report how incentives can address or create inequalities; enhance or diminish intrinsic motivation and wellbeing, and how they may have a positive or negative impact on relationships within their personal networks and/or health providers. While incentives may raise awareness and direct service delivery to areas of need, this may be detrimental to other areas of health care. This detailed exposition thereby provides new insights into positive and negative consequences, as well as why, how and for whom these consequences might occur.

The strengths of this study include the mixed methods, multi-disciplinary, three site approach, which enabled us to engage with consumers and providers with and without direct experience of incentive interventions and the target behaviours. Data collection was undertaken by five researchers over a prolonged period of time. This resulted in variations in terms of how the interview and focus group questions were framed. Access to more disadvantaged settings and sampling techniques enabled us to obtain a broad range of views from participants with diverse socio-economic and behaviour characteristics, including participants who seldom engage in health services research. The limitations relate to where only restricted views were collected and the characteristics of the sample, despite every effort to recruit “harder to reach” participants. The White ethnic study population (88.6% of women/significant others and 89.3% of survey respondents) is slightly higher than census data for England (80.8%) [46] and Scotland (92.9%) [47]. In addition a much higher percentage of the women/significant others were married (77.3%) compared with rates for England in 2011 of (46.6%) [48]. A large percentage of the survey participants had never smoked (74.5%), which would be expected for health professionals. Furthermore only a small number of the women reported that they had not tried to breastfeed or planned to formula feed, perhaps reflecting that 80% of UK women initiate breastfeeding [49] or desirable response bias. In addition, limited views from wider family members were collected. Researchers selected the study vignettes from the evidence syntheses and drafted the shortlist of incentive strategies to frame the incentives. However, with service-user co-applicant input vignettes were also a strength, as the concrete scenarios enabled participants to highlight issues from a more individualised, reflective perspective than would have been achieved using more abstract question–answer techniques. A further limitation may relate to how the interviews/focus groups asked individuals to reflect on ‘consequences’ of incentives; whereas the survey specifically asked individuals to report on positive and negative consequences. In the light of our findings, more focused topic guides may well elicit additional insights, and this should be addressed within future studies. The limitations of the health professional survey are discussed fully elsewhere [14]. In particular, the free text question followed specific Likert Scale questions about agreement with our shortlist of seven most promising financial incentive strategies, which included shopping vouchers, a free breast pump and provider payments for meeting targets. Therefore it is possible that the free text responses could have been interpreted in the context of responses to the earlier questions. As non-response bias is also a concern, the free text data was only used for triangulation purposes.

Our findings on incentives to service providers support those previously reported for a wider range of health outcomes, in terms of how incentives can narrow health equalities through facilitating access to healthcare [4], [26]. While universal provision of incentives to consumers could guard against a postcode lottery of care, targeted support was important to engage those most at need and address embedded social norms. However, we identified the potential for differential uptake across social classes and the potential for health inequalities to increase, as noted for other lifestyle behaviour change interventions [50]. Of concern a MORI survey of the public acceptability of our shortlist of incentive strategies conducted as part of the BIBS study revealed important differences in attitudes between the more and less educated, and between women and men [14], [51]. Women compared to men were more likely to disagree with shopping voucher incentives for smoking cessation or breastfeeding, and those with lower levels of education, a reliable proxy for disadvantage [52], disagreed more with smoking cessation incentives and a breast pump [14], [51]. The assumption of governments that incentives will forge partnerships to deliver better health outcomes, reduce health inequalities and build social capital requires further testing.

At an individual level, incentives were the tipping point for some women, the opportunity that facilitated change [53]. However, as reported by others, concerns were raised that incentives could “crowd out” intrinsic motivation [4], [6], [7]. As incentives were identified to have the potential to enhance wellbeing, and wellbeing is identified as an important driver in behaviour-related infant feeding decisions [54], preferences for incentives that increase positive affect and add value for women as connectors for continued support [29] rather than diminish intrinsic motivation should be considered. How the consequences of incentives for behaviour change might impact either positively or negatively beyond the individual to social networks, communities and engagement within services is uncertain.

Media debates on the use of financial incentives have tended to be negative [55], [56]. However, a UK population based study by Promberger and colleagues to assess the acceptability of incentive-based treatments for smoking cessation and weight loss reported that the acceptability of financial incentives is not necessarily negative but rather contingent on the target behaviour, the type of incentive and their effectiveness [57]. Whilst positive unintended consequences have been reported in the literature [4], [18], [28], [29] our study also illustrates positive consequences that have received little attention to date. Autonomy is of particular note where incentives are perceived as bribes which can undermine free will and reflect a ‘*nanny state*’ resulting in diminished individual responsibility for health choices [58]. In contrast, consumers and professionals in our study reported that unrestricted vouchers can promote individual autonomy for the most disadvantaged through providing a rare opportunity for choice and self-reward. Similarly discourses of incentives as ‘unfair’ for rewarding ‘bad behaviour’, of discouraging individual responsibility or for targeting only disadvantaged communities contrast with narratives of feeling valued, more confident and improved self-esteem. Our interpretation is that media debates on the advantages and disadvantages of financial incentives can be easily biased towards intellectual philosophical, political and ethical arguments about the role of the state, without considering the perspectives of more disadvantaged families who are struggling to do the best for their children. This is particularly important as children have no choice and public acceptability is greatest for incentives to protect their wellbeing [2].

Related issues of ‘gaming and cheating’ were reported in terms of duplicitous activities amongst consumers and providers [17], [18], [25], [26], particularly when verifiable ‘proof’ of these behaviours was difficult to obtain. As incentives of higher values are believed to have a stronger correlation associated with unintended consequences [59], [60] consideration of the value and type (e.g. financial or behavioural) to mitigate against gaming behaviours appears crucial. The potential positive and negative impact of incentive schemes on provider–women relationships in our study are evident in the literature: increased access and improved rapport [29]; mistrust and alienation [4], [30] and limiting support for non-target behaviours [4], [5]. These findings highlight the need for sensitive, authentic, person-centred communication [61] as women dislike feeling judged or pressurised to behave in a way deemed appropriate by others [54], [62].

While incentives may have the potential to create shared aims, oppositional views emerged with regard to the impact of incentives on other health outcomes and service delivery. From one perspective, the investment in incentives would enable specialist and targeted services to be developed as reported by Cahill & Petera [18] and Gillam et al. [4]. However, the recent economic downturn led to views that investment in incentives was impractical, unethical and immoral, as well as creating expectations of ‘payments’ for other health-related behaviours [26]. Contentions largely centred on potential opportunity costs. However, in line with key government priorities of health promotion and prevention, a pro-active approach and associated funding to address these pervasive health behaviours were believed to have long-term benefits of reduced health costs, staff time and absenteeism rates.

A checklist has been developed to help decision makers assess when incentives might do more good than harm, to help prevent premature or inappropriate implementation [28]. This checklist highlights some of the key areas of contention in terms of ascertaining a) effectiveness of incentives prior to roll-out; b) appropriate targeting and eligibility criteria; c) valid and independent verification of behaviour outcomes; d) the implications of incentives on behaviours and motivation and e) that the benefits outweigh the potential for negative consequences in relation to ‘attention shift’ in terms of decreasing efforts in other health-care areas; ‘gaming’ behaviours and implications for the provider-consumer relationship. While this checklist highlights the need for systems and structures to be in place to prevent against negative consequences, our study emphasises how unintended positive consequences of incentive provision also require consideration.

## Conclusion

The utility and acceptability of incentive provision is a controversial area, which can generate emotive and oppositional responses. Assumptions that incentives will help to address health inequalities, increase the reach of behaviour change interventions and facilitate a cultural shift towards desired behaviours require rigorous testing given the conflicting narratives around consequences. Prospective mixed methods approaches at the incentive intervention design and feasibility stages are needed, together with consideration of potential unintended consequences at all levels of service provision: for the participants, the population and those delivering the incentives and services. Process evaluations are also needed to capture the unintended consequences during a trial, with longer term follow up of key areas of concern after a trial has ended.

When planning an incentive intervention, care should be taken to assess discipline bias (e.g. philosophical, political, ethical, health) in narratives around personal autonomy, the role of the state and responsibility. These can be contrasted to personal narratives from less often heard voices representing the target population that incentives aim to help. Anticipation of positive and negative unintended consequences should be integral to the planning, design and implementation of interventions that include incentives, helping to ensure that any benefits are maximised.

## Supporting Information

Appendix S1 **Web Survey for Early Years Professionals.**
(PDF)

## References

[1] Marteau TM, Ashcroft RE, Oliver A (2009) Using financial incentives to achieve healthy behaviour. BMJ 338: b1415. 19359291 10.1136/bmj.b1415

[2] Diepeveen SL, Ling T, Suhrcke M, Roland M, Marteau T (2013) Public acceptability of government intervention to change health-related behaviours: A systematic review and narrative synthesis. BMC Public Health 13: 756 10.1186/1471-2458-13-756 23947336 PMC3765153

[3] Giles ER, S, McColl E, Sniehotta FF, Adams J (2014) The Effectiveness of Financial Incentives for Health Behaviour Change: Systematic Review and Meta-Analysis. PLoS ONE 9: e90347. 24618584 10.1371/journal.pone.0090347 PMC3949711

[4] Gillam SJ, Siriwardena AN, Steel N (2012) Pay-for-performance in the United Kingdom: impact of the quality and outcomes framework: a systematic review. Ann Fam Med 10: 461–468. 22966110 10.1370/afm.1377 PMC3438214

[5] Flodgren G, Eccles MP, Shepperd S, Scott A, Parmelli E, et al (2011) An overview of reviews evaluating the effectiveness of financial incentives in changing healthcare professional behaviours and patient outcomes. Cochrane Database Syst Rev Issue 7: CD009255. 10.1002/14651858.CD009255 PMC4204491 21735443

[6] Frey B (1997) Not just for the money: An economic theory of personal motivation. Brookfield, VT: Edward Elgar Publishing.

[7] Deci EL, Koestner R, Ryan RM (1999) A meta-analytic review of experiments examining the effects of extrinsic rewards on intrinsic motivation. Psychol Bull 125: 627–668. 10589297 10.1037/0033-2909.125.6.627

[8] Johnston M, Sniehotta F (2010) Financial incentives to change patient behaviour. J Health Serv Res Policy 15: 131–132. 20555040 10.1258/jhsrp.2010.010048

[9] Fiszbein AN, Schady N, Schady N, Ferreira FG, Grosh M, et al. (2009) Conditional Cash Transfers: Reducing Present and Future Poverty. A World Bank Policy Research Report. 47603. Available: https://openknowledge.worldbank.org/bitstream/handle/10986/2597/476030PUB0Cond101Official0Use0Only1.pdf?sequence=1. Accessed 2013 Sep 5.

[10] Merton RK (1936) The Unanticipated Consequences of Purposive Social Action. Am Sociol Rev 1: 894–904.

[11] Free Yourself - Smoking, It’s Not Worth It! (Tayside Quit4U Scheme). Available: http://www.canstopsmoking.com/local-help/free-yourself-smoking,-it’s-not-worth-it!-(tayside-quit4u-scheme). Accessed 2013 Oct 13.

[12] Encouraging Teenage Mothers to Breastfeed. Available: http://www1.uwe.ac.uk/bl/research/bsmc/researchprojects/healthandexercise/encouragingbreastfeeding.aspx. Accessed 2013 Sep 5.

[13] Breastfeeding mothers offered £200 in shop vouchers. Available: http://www.bbc.co.uk/news/health-24900650. Accessed 2013 Nov 12.

[14] Morgan H, Hoddinott P, Thomson G, Crossland N, Farrar S, et al. (in press) Benefits of incentives for breastfeeding and smoking cessation in pregnancy (BIBS): a mixed methods study to inform trial design. Health Technology Assessment http://www.nets.nihr.ac.uk/projects/hta/103102. 10.3310/hta19300 PMC4780978 25897655

[15] Hoddinott P, Hislop J, Morgan H, Stewart F, Farrar S, et al (2013) Incentive interventions for smoking cessation in pregnancy: a mixed methods evidence synthesis. Lancet 380 (Suppl (3)) S48.

[16] Cahill K, Perera R (2008) Competitions and incentives for smoking cessation. Cochrane Database Syst Rev Issue 3: CD004307. 10.1002/14651858.CD004307.pub3 18646105

[17] Cahill K, Perera R (2011) Competitions and incentives for smoking cessation. Cochrane Database Syst Rev Issue 4: CD004307. 10.1002/14651858.CD004307.pub4 21491388

[18] Cahill K, Perera R (2008) Quit and Win contests for smoking cessation. Cochrane Database Syst Rev Issue 4: Art. No: CD004986. 10.1002/14651858.CD004986.pub3 18843674

[19] National Institute of Health and Clinical Excellence (2010) The use of incentives to improve health: Citizens Council Meeting. London, National Institute for Health and Clinical Excellence.

[20] Cinciripini PM, Blalock JA, Minnix JA, Robinson JD, Brown VL, et al (2010) Effects of an Intensive Depression-Focused Intervention for Smoking Cessation in Pregnancy. J Consult Clin Psychol 78: 44–54. 20099949 10.1037/a0018168 PMC2881321

[21] Van Herck P, De Smedt D, Annemans L (2010) Systematic review: effects, design choices, and context of pay-for-performance in health care. BMC Health Serv Res 10: 247. 20731816 10.1186/1472-6963-10-247 PMC2936378

[22] Kavanagh J, Trouton A, Oakley A, Powell C (2006) A systematic review of the evidence for incentive schemes to encourage positive health and other social behaviours in young people. Available: http://eppi.ioe.ac.uk/cms/LinkClick.aspx?fileticket=Soskzt1sFZs%3D&tabid=671&mid=1562. Available 2013 Feb 12.

[23] Radley A, Ballard P, Eadie D, MacAskill S, Donnelly L, et al (2013) Give It Up for Baby: Outcomes and factors influencing uptake of a pilot smoking cessation incentive scheme for pregnant women. BMC Public Health 13: 343 10.1186/1471-2458 23587161 PMC3640949

[24] Kavanagh J, Stansfield C, Thomas J (2009) Incentives to improve smoking, physical activity, dietary and weight management behaviours: a scoping review of the research evidence. Available: https://eppi.ioe.ac.uk/cms/Default.aspx?tabid=2456. Accessed 2013 Feb 12.

[25] Kane RL, Johnson PE, Town RJ, Butler M (2004) Economic incentives for preventive care. Evidence Report: Technology Assessment (Summary) 101: 1–7. PMC4781426 15526397

[26] Scott A, Sivey P, Ait OD, Willenberg L, Naccarella L, et al (2011) The effect of financial incentives on the quality of health care provided by primary care physicians. Cochrane Database Syst Rev Issue 9: CD008451. 10.1002/14651858.CD008451.pub2 21901722

[27] Paul-Ebhohimhen V, Avenell A (2008) Systematic review of the use of financial incentives in treatments for obesity and overweight. Obes Rev 9: 355–367. 17956546 10.1111/j.1467-789X.2007.00409.x

[28] Glasziou PP, Buchan H, Del M, Doust J, Harris M, et al (2012) When financial incentives do more good than harm: a checklist. BMJ 345: e5047. 22893568 10.1136/bmj.e5047

[29] Thomson G, Dykes F, Hurley MA, Hoddinott P (2012) Incentives as connectors: insights into a breastfeeding incentive intervention in a disadvantaged area of North-West England. BMC Pregnancy Childbirth 12: 22 10.1186/1471-2393-12-22 22458841 PMC3414740

[30] Coleman T (2010) Do financial incentives for delivering health promotion counselling work? Analysis of smoking cessation activities stimulated by the quality and outcomes framework. BMC Public Health 10: 167 10.1186/1471-2458-10-167 20346154 PMC3091543

[31] Edinburgh: Information Statistics Division. Breastfeeding statistics financial year 2011/12. NHS National Services Scotland. Available: http://www.isdscotland.org/Health-Topics/Child-Health/Infant-Feeding/. Accessed 2013 Oct 12.

[32] Edinburgh: Information Statistics Division. Data tables: births in Scottish hospitals - Year ending 31st March 2012. NHS National Services Scotland. Available: http://www.isdscotland.org/Health-Topics/Maternity-and-Births/Publications/data-tables.asp. Accessed 2013 Oct 12.

[33] Breastfeeding by NHS Board of Review: Financial Years 2001/02–2011/12. Edinburgh: Information Statistics Division, NHS National Services Scotland. Available: http://www.isdscotland.org/Health-Topics/Child-Health/Publications/data-tables.asp. Accessed 2013 Oct 12.

[34] Tappin DM, Bauld L, Tannahill C, de Caestecker L, Radley A, et al (2012) The cessation in pregnancy incentives trial (CPIT): study protocol for a randomized controlled trial. Trials 13: 113 10.1186/1745-6215-13-113 22818493 PMC3482147

[35] Child and Maternal Health Intelligence Network. Breastfeeding profiles. London, Public Health Available: http://atlas.chimat.org.uk/IAS/dataviews/report?reportId=351&viewId=355&geoReportId=3198&geoId=4&geoSubsetId=. Accessed 2013 Oct 2.

[36] Local tobacco control profiles for England. London, Public Health England. Available: http://www.tobaccoprofiles.info/tobacco-control#gid/1000110/par/E12000002/ati/102/page/0. Accessed 2013 Oct 2.

[37] Chamberlain LB, McMahon M, Philipp BL, Merewood A (2006) Breast pump access in the inner city: A hospital-based initiative to provide breast pumps for low-income women. J Hum Lact 22: 94–98. 16467290 10.1177/0890334405284226

[38] Gulliver SB, Colby SM, Hayes K, Raffa SD (2004) Tobacco cessation treatment for pregnant smokers: incorporating partners and incentives. Med Health R I 87: 9–12. 14989074

[39] Heil SH, Higgins ST, Bernstein IM, Solomon LJ, Rogers RE, et al (2008) Effects of voucher-based incentives on abstinence from cigarette smoking and fetal growth among pregnant women. Addict 103: 1009–1018. 10.1111/j.1360-0443.2008.02237.x PMC2731575 18482424

[40] Pugh LC, Milligan RA (1998) Nursing intervention to increase the duration of breastfeeding. Appl Nurs Res 11: 200–194. 10.1016/s0897-1897(98)80318-2 9852662

[41] Volpe EM, Bear M (2000) Enhancing breastfeeding initiation in adolescent mothers through the Breastfeeding Educated and Supported Teen (BEST) Club. J Hum Lact 16: 196–200. 11153152 10.1177/089033440001600303

[42] Walsh RA, Redman S, Brinsmead MW, Byrne JM, Melmeth A (1997) A smoking cessation program at a public antenatal clinic. Am J Public Health 87: 1201–1204. 9240113 10.2105/ajph.87.7.1201 PMC1380897

[43] Cattaneo A, Borgnolo G, Simon G (2001) Breastfeeding by objectives. Eur J Publ Health 11: 397–401. 10.1093/eurpub/11.4.397 11766480

[44] Ritchie J, Spencer L (1994) Qualitative data analysis for applied policy research. In: Bryman A, Burgess RG, editors. Analyzing qualitative data. London: Routledge. pp. 173–194.

[45] Braun V, Clarke V (2006) Using thematic analysis in psychology. Qual Res. Psychol 3(2): 77–101.

[46] Office for National Statistics (2011) Available: http://www.ons.gov.uk/ons/dcp171778_292378.pdf. Accessed 2014 Jul 30.

[47] Scotland’s Census (2011) Available: http://www.scotlandscensus.gov.uk/. Accessed 2014 Jul 30.

[48] Office for National Statistics (2011) Available: http://www.ons.gov.uk/ons/dcp171778_290685.pdf. Accessed 2014 Jul 30.

[49] McAndrew F, Thompson J, Fellows L, Large A, Speed M, et al. (2012) Infant feeding survey 2010: summary. Available: http://www.hscic.gov.uk/catalogue/PUB08694. Accessed 2013 Aug 20.

[50] Jepson RG, Harris FM, Platt S, Tannahill C (2010) The effectiveness of interventions to change six health behaviours: a review of reviews. BMC Public Health 10: 538 10.1186/1471-2458-10-538 20825660 PMC2944371

[51] Hoddinott P, Morgan H, MacLennan G, Sewel K, Thomson G, et al (2014) Public acceptability of financial incentives for smoking cessation in pregnancy and breastfeeding. BMJ Open 4: e005524. 10.1136/bmjopen-2014-005524 PMC4120368 25037645

[52] Marmot M (2004) Status Syndrome. London: Bloomsbury.

[53] Thaler RH, Sunstein CR (2008) Nudge: improving decisions about health, wealth, and happiness. New Haven: Yale University Press.

[54] Hoddinott P, Craig L, Britten J, McInnes R (2012) A serial qualitative study of infant feeding experiences: idealism meets realism. BMJ Open 2: e000504. 10.1136/bmjopen-2011-000504 PMC3307036 22422915

[55] Reward healthy people with tax rebates to save the NHS, urges thinktank. Available: http://www.theguardian.com/society/2014/may/01/nhs-reward-healthy-with-tax-rebates. Accessed 2014 Jun 20.

[56] Park JD, Mitra N, Asch DA (2012) Public opinion about financial incentives for smoking cessation. Preventive Med 55 (Suppl): S41–S45. 10.1016/j.ypmed.2012.06.013 22735040

[57] Promberger M, Dolan P, Marteau TM (2012) “Pay them if it works”: Discrete choice experiments on the acceptability of financial incentives to change health related behaviour. Soc Sci Med 75: 2509–2514. 23102753 10.1016/j.socscimed.2012.09.033 PMC3686527

[58] Ashcroft RE (2011) Personal financial incentives in health promotion: where do they fit in an ethic of autonomy? Health Expect 14: 191–200. 21348904 10.1111/j.1369-7625.2011.00664.x PMC3123700

[59] Doran T, Fullwood C, Kontopantelis E, Reeves D (2008) Effect of financial incentives on inequalities in the delivery of primary clinical care in England: analysis of clinical activity indicators for the quality and outcomes framework. Lancet 372: 728–736. 18701159 10.1016/S0140-6736(08)61123-X

[60] Sutton M, Elder R. Guthrie B, Watt G (2010) Record rewards: the effects of targeted quality incentives on the recording of risk factors by primary care providers. Health Econ 19: 1–13. 10.1002/hec.1440 19206084

[61] Schmied V, Beake S, Sheehan A, McCourt C, Dykes F (2011) Women’s perceptions and experiences of breastfeeding support: a metasynthesis. Birth 38: 49–60. 21332775 10.1111/j.1523-536X.2010.00446.x

[62] Graham H, Sowden A, Flemming K, Heirs M, Fox D (2012) Using qualitative research to inform interventions to reduce smoking in pregnancy in England: a systematic review of qualitative studies: Public Health Research Consortium. Available: http://phrc.lshtm.ac.uk/project_2005-2011_a810.html. Accessed 2013 Feb 12.

