# Supplementary material for: Unintended Consequences of Incentive Provision for Behaviour Change and Maintenance around Childbirth
Source: PLoS One. 2014 Oct 30;9(10):e111322. doi: 10.1371/journal.pone.0111322 (PMC4214733; doi:10.1371/journal.pone.0111322)
Supplement: Appendix S1 — Web Survey for Early Years Professionals. (PDF) [file pone.0111322.s001.pdf]

***1. Stopping smoking in pregnancy benefits the health of the baby and the mother. Research shows that providing shopping vouchers to women who prove that they have stopped smoking during pregnancy increases the number of women who stop. While some people feel that providing vouchers is appropriate, others feel that it is wrong or unfair.**

Do you agree or disagree that shopping vouchers should be provided to women who prove that they have stopped smoking during pregnancy?

- ◯ Strongly agree
- ◯ Tend to agree
- ◯ Neither agree nor disagree
- ◯ Tend to disagree
- ◯ Strongly disagree

***2. What is the highest amount of shopping voucher you think it would be acceptable to provide a woman who proves that she has stopped smoking during pregnancy?**

- ◯ £2 per month
- ◯ £10 per month
- ◯ £20 per month
- ◯ £40 per month
- ◯ £60 per month
- ◯ £80 per month

***3. Do you think that it is acceptable to provide shopping vouchers to women who prove that they have stopped smoking during pregnancy, regardless of their income, or only to women on low incomes?**

- ◯ To all women, regardless of income
- ◯ Only to women on low incomes

***4. Some women start smoking again after the birth of their baby, particularly if their partner or someone at home smokes. Please tell us whether you agree or disagree with each of the following statements.**

	Strongly agree	Tend to agree	Neither agree nor disagree	Tend to disagree	Strongly disagree
It is acceptable to provide shopping vouchers to a woman for two months after the birth of her baby if she proves that she is still not smoking	◯	◯	◯	◯	◯
It is acceptable to provide shopping vouchers to a woman for two months after the birth of her baby if she never lets anyone smoke in her home	◯	◯	◯	◯	◯

***5. Do you agree or disagree that local health services should receive additional funding if they reach targets for the number of women who prove that they have stopped smoking during pregnancy?**

- Strongly agree
- Tend to agree
- Neither agree nor disagree
- Tend to disagree
- Strongly disagree

***6. Breastfeeding benefits the health of the baby and the mother. While some people feel it is appropriate to provide shopping vouchers to encourage breastfeeding, other people feel it is wrong or unfair.**

Do you agree or disagree that shopping vouchers should be provided to women who breastfeed for the first six months after the birth of their child?

- ◯ Strongly agree
- ◯ Tend to agree
- ◯ Neither agree nor disagree
- ◯ Tend to disagree
- ◯ Strongly disagree

***7. What is the highest amount of shopping voucher you would consider acceptable for women who breastfeed?**

- ◯ £2 per month
- ◯ £10 per month
- ◯ £20 per month
- ◯ £40 per month
- ◯ £60 per month
- ◯ £80 per month

***8. Do you agree or disagree that shopping vouchers should be provided to all women who breastfeed, regardless of their income, or only to women on low incomes?**

- ◯ To all women, regardless of income
- ◯ Only to women on low incomes

***9. Do you agree or disagree that local health services should receive additional funding if they reach targets for the number of women who breastfeed?**

- ◯ Strongly agree
- ◯ Tend to agree
- ◯ Neither agree nor disagree
- ◯ Tend to disagree
- ◯ Strongly disagree

***10. Some women who breastfeed like to express milk. This means that they can get breast milk when mother and baby are apart.**

To express milk, some women find a breast pump useful. Women can buy breast pumps ranging from £20 to over £100. Do you agree or disagree that a breast pump costing around £40 should be available for free on the NHS, to help women to continue breastfeeding?

- ◯ Strongly agree
- ◯ Tend to agree
- ◯ Neither agree nor disagree
- ◯ Tend to disagree
- ◯ Strongly disagree

***11. Do you have any children? Please include any children who are grown up now, and any children who do not live with you.**

- ◯ Yes
- ◯ No

***12. Have any of your children ever been breastfed or received breast milk, even if only for a day or two?**

- ◯ Yes
- ◯ No

*13. Do you currently smoke or have you ever smoked?

- ◯ Yes, I currently smoke every day
- ◯ Yes, I currently smoke, but not every day
- ◯ Yes, I used to smoke but have quit
- ◯ No, I have never smoked
- ◯ I prefer not to answer

*14. Have you ever tried to stop smoking?

- ◯ Yes
- ◯ No

15. We would like you to imagine that your local health service is going to run a scheme that provides incentives for stopping smoking in pregnancy. What do you think the consequences might be for participants and/or staff?

Positive consequences	
Negative consequences	

16. Similarly, we would like you to imagine that your local health service is going to run a scheme that provides incentives for breastfeeding. What do you think the consequences might be for participants and/or staff?

Positive consequences

Negative consequences

Some questions about your work

***17. In which country are you based?**

- ◯ England
- ◯ Scotland

*18. What is your job or role?

- Midwife
- Other maternity nursing or caring staff (e.g. nursery nurse, RGN)
- Health visitor
- Health visitor assistant
- Paediatrician
- Obstetrician
- General practitioner
- Practice nurse
- Public health doctor or practitioner
- Manager of the above
- Student (please specify below)
- Other (please specify below)

My job or role is:

Some questions about you

*19. Are you male or female?

- ◯ Male
- ◯ Female
- ◯ Prefer not to say

***20. Which category below includes your age?**

- ◯ 16-24
- ◯ 25-34
- ◯ 35-44
- ◯ 45-54
- ◯ 55-64
- ◯ 65 and over
- ◯ Prefer not to say

*21. Please describe your ethnicity

- ◯ White - White
- ◯ White - British
- ◯ White - Irish
- ◯ White - Other White background
- ◯ Mixed - White and Black Caribbean
- ◯ Mixed - White and Black African
- ◯ Mixed - White and Asian
- ◯ Mixed - Other Mixed background
- ◯ Asian or Asian British - Indian
- ◯ Asian or Asian British - Pakistani
- ◯ Asian or Asian British - Bangladeshi
- ◯ Asian or Asian British - Other Asian background
- ◯ Black or Black British - Caribbean
- ◯ Black or Black British - African
- ◯ Black or Black British - Other Black background
- ◯ Black or Black British - All Black Groups
- ◯ Chinese or Other Ethnic Groups - Chinese
- ◯ Chinese or Other Ethnic Groups - Other Ethnic Group
- ◯ Prefer not to say

***22. We would like to invite you to participate in a short 15 minute telephone interview to tell us more about your views. If you are interested, we will request your consent to record the interview, which will be confidential and anonymous. Would you be interested in being interviewed?**

- ◯ No, thank you
- ◯ Yes, and I have entered my contact details below

My email address and contact telephone number (mobile preferred) are:

***23. THANK YOU FOR PARTICIPATING IN OUR SURVEY**

40 x £5 Amazon (www.amazon.co.uk) vouchers are available to be won. To enter, please type your email address below. Please note that your email address will be used for the prize draw only and you will not be contacted by researchers again unless you expressed an interest in being interviewed on the previous page.

- ◯ No, thank you. Please take me to the end of the survey.
- ◯ I would like to enter the prize draw and my email address is:
